# Automatic image registration on intraoperative CBCT compared to Surface Matching registration on preoperative CT for spinal navigation: accuracy and workflow

**DOI:** 10.1007/s11548-024-03076-4

**Published:** 2024-02-20

**Authors:** Henrik Frisk, Gustav Burström, Oscar Persson, Victor Gabriel El-Hajj, Luisa Coronado, Susanne Hager, Erik Edström, Adrian Elmi-Terander

**Affiliations:** 1 Department of Clinical Neuroscience, Karolinska Institutet, 171 77 Stockholm, Sweden; 2 Clinical Affairs, Brainlab AG, Munich, Germany; 3 Capio Spine Center Stockholm, Löwenströmska Hospital, Upplands-Väsby, Sweden; 4 Department of Surgical Sciences, Uppsala University, Uppsala, Sweden

**Keywords:** Patient tracking, Reference frame, Surface Matching, CBCT, Spine surgery, Surgical navigation

## Abstract

**Introduction:**

Spinal navigation solutions have been slower to develop compared to cranial ones. To facilitate greater adoption and use of spinal navigation, the relatively cumbersome registration processes need to be improved upon. This study aims to validate a new solution for automatic image registration and compare it to a traditional Surface Matching method.

**Method:**

Adult patients undergoing spinal surgery requiring navigation were enrolled after providing consent. A registration matrix—Universal AIR (= Automatic Image Registration)—was placed in the surgical field and used for automatic registration based on intraoperative 3D imaging. A standard Surface Matching method was used for comparison. Accuracy measurements were obtained by comparing planned and acquired coordinates on the vertebrae.

**Results:**

Thirty-nine patients with 42 datasets were included. The mean accuracy of Universal AIR registration was 1.20 ± 0.42 mm, while the mean accuracy of Surface Matching registration was 1.94 ± 0.64 mm. Universal AIR registration was non-inferior to Surface Matching registration. Post hoc analysis showed a significantly greater accuracy for Universal AIR registration. In Surface Matching, but not automatic registration, user-related errors such as incorrect identification of the vertebral level were seen.

**Conclusion:**

Automatic image registration for spinal navigation using Universal AIR and intraoperative 3D imaging provided improved accuracy compared to Surface Matching registration. In addition, it minimizes user errors and offers a standardized workflow, making it a reliable registration method for navigated spinal procedures.

**Supplementary Information:**

The online version contains supplementary material available at 10.1007/s11548-024-03076-4.

## Introduction

Over the past 30 years, spine surgery has undergone a rapid transformation largely due to technological advances in image-guided navigation. In traditional open, freehand, instrumented surgery, the screw trajectory is estimated after exposing the screw entry point and the nearby relevant anatomical landmarks. This is to ensure that screws are correctly placed and injury to neurovascular structures is avoided. The freehand technique requires large incisions and causes significant tissue damage. In trauma or deformity surgery when the anatomy is altered, the normal trajectories can be distorted increasing the risk of screw misplacement. 3-D navigational techniques were introduced after the development of mobile fan-beam and cone-beam computed tomography (CT) devices and computers with fast processor speed and navigational software. 3-D real-time rendering simplifies the conceptualization of 3-D anatomy and offers a high degree of accuracy. The adaptation and evolution of image-guided navigation techniques have allowed a move toward minimally invasive surgery (MIS) where the lack of anatomical visualization can be compensated for with navigation [1]. Several reports have shown an improved workflow and increases in the safety, accuracy, and efficiency of MIS procedures [2–6]. However, the development of spinal navigation solutions has been slower than the cranial ones. The relatively complicated and time-consuming registration process of spinal navigation devices and the inherent risk for decreased accuracy with distance from the dynamic reference frame may provide part of the explanation [4, 7, 8]. Despite efforts to implement novel registration and tracking methods [9, 10], the traditional infrared, reference-based, outside-in, navigation devices still dominate the market. Commercially available navigation devices use an indirect method for spine tracking where optical hardware is used to recognize a dynamic reference frame with reflecting spheres in a predetermined geometrical pattern [11–13]. Tracking is initiated by providing the navigation system with information on the position of the patient either through a user feedback procedure to match the patient’s anatomy to the preoperative images, or by using intraoperative automatic image registration [14, 15]. The manual registration typically requires several minutes to complete the multi-step user feedback procedure necessary to achieve the required accuracy [16–19]. Due to manual errors, or factors such as movement due to respiration, the registration procedure may need to be repeated before an acceptable accuracy is achieved. Repeating the registration during surgery may not be feasible. Thus, manual registration prolongs and complicates the surgical process, limiting surgical efficiency and the wider implementation of spinal navigation. These shortcomings can be improved by the automatic detection and registration of landmarks [20].

However, automatic intraoperative image registration has only been possible when the imaging device and the navigation system share a common interface with integrated calibration [14, 15].

The aim of this study was to validate a novel automatic registration solution designed to be independent of the 3D scanning device: Universal AIR (Brainlab AG, Munich, Germany), and compare its workflow and accuracy to a traditional Surface Matching registration method (Spine and Trauma Navigation and Registration SW, Brainlab AG, Munich, Germany).

## Materials and methods

### Study design

This is a prospective, interventional, non-randomized, study with consecutive patient enrollment to compare two different registration methods for surgical navigation in the spine. The study hospital is a publicly funded and owned tertiary care center serving a region of roughly 2.3 million inhabitants and the only neurosurgical center in the region. The study cohort consisted of adult patients (≥ 18 years) who were surgically treated for any spinal disorder requiring surgical navigation at the study center between 2020 and 2022. Patients were not eligible if a preoperative CT scan suitable for navigation was not available. Signed informed consent was obtained from all participants. The study was approved by the National Ethical Review Board (Dnr: 2020-06541).

### Workflow

#### Surgical Navigation

**Fig. 1:**
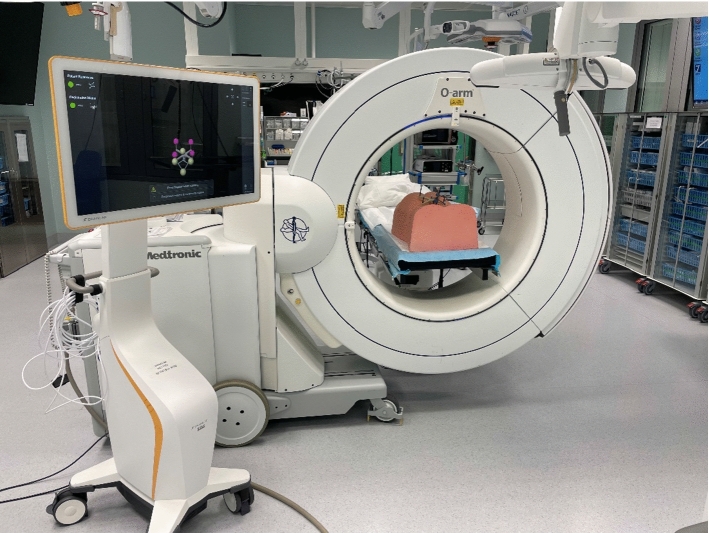
The navigation setup in the OR with the O-arm scanner and Brainlab curve navigation

The navigated spine surgeries were performed in a conventional OR equipped with a navigation system with dedicated spine navigation software (Brainlab, Curve1.2 Dual Navigation Station, Software: Spine &Trauma 3D Navigation 1.5. Registration Software Spine Surface Matching 1.0. Automatic Registration 2.5), and an O-arm scanner (Medtronic, USA). The OR table is placed centrally in the room, and the O-arm positioned perpendicularly to the table so that the gantry is moved easily over the table for imaging. The camera of the navigation system must be placed so that it has an uninterrupted line of sight to identify all marker spheres of the dynamic reference frame and, for automatic registration, the Universal Air device (Fig. 1).

### Surgical technique

All patients were under general anesthesia and placed in the prone position with the head fixed in a Mayfield clamp (cervical and upper thoracic cases) or on a pillow (lower thoracic and lumbar cases). Prior to surgery, the identification of the spinous process of the vertebra adjacent to the pathology in thoracic and lumbar cases was accomplished using computed tomography guidance and marking the level with injection of a sterile carbon suspension. In contrast, intraoperative fluoroscopy was used for vertebral identification in the cervical spine. A posterior midline approach for an open surgery was performed in all cases. The spinous processes and laminae of the vertebrae of interest were exposed. One vertebra in the surgical field was selected for registration, and a dynamic reference frame (carbon fiber clamp with reference array) was attached to the spinous process. Three micro-screws were inserted into the laminae of that vertebra, placing the screws as far apart as possible. The purpose of placing the screws was to create a reliable and accurate reference for error measurement of the registration method. The screws are ideal landmarks because of their fixed position and easily recognizable heads with a well-defined center. The screws were planned on the intraoperative CT scan, and their actual coordinates were acquired with a pointer.

### Surface Matching

**Fig. 2:**
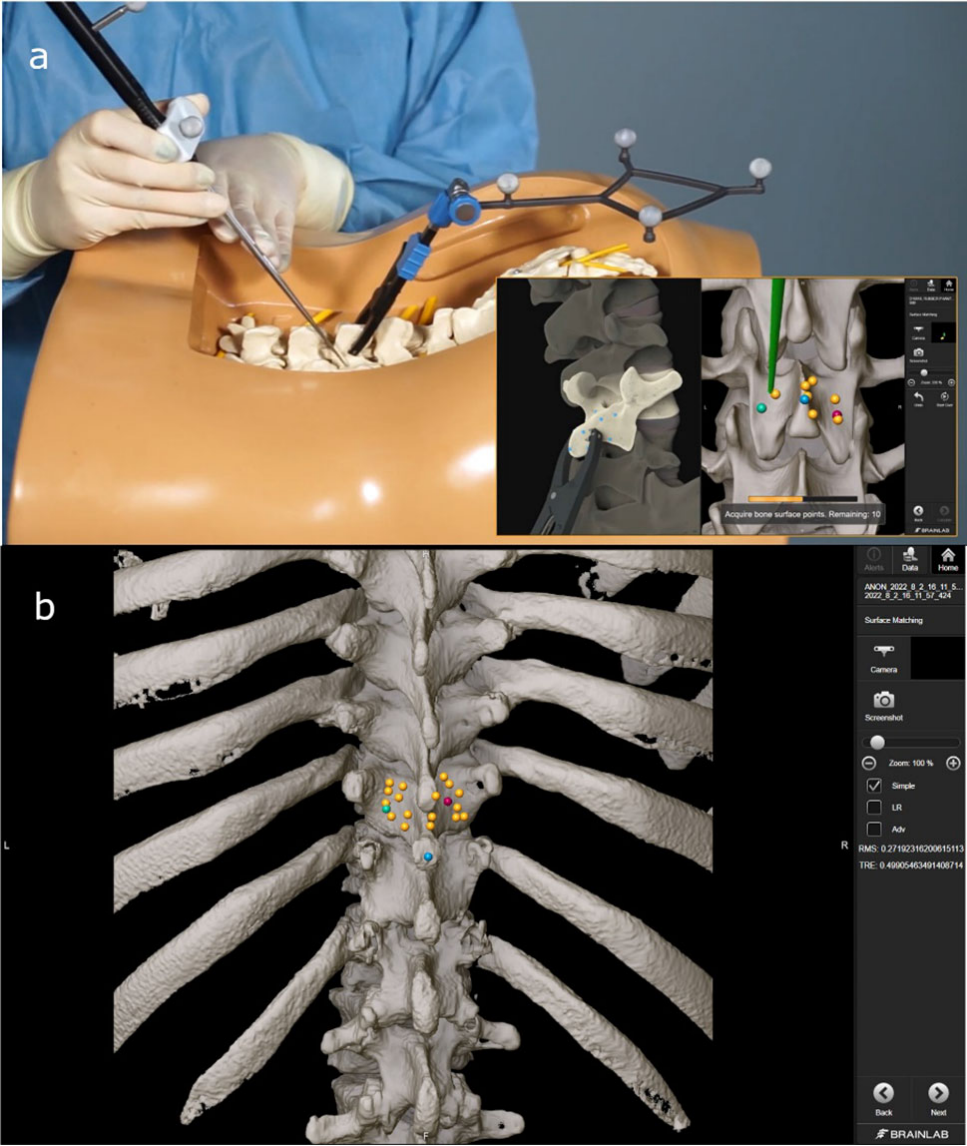
**a** Demonstration of Surface Matching on a phantom. **b** Acquired points during Surface Matching registration as displayed in the navigation software. The initial points that serve to identify the correct vertebra and its orientation are seen in blue, red and green. The yellow points indicate the subsequent Surface Matching positions.

The preoperative CT scan was imported to the navigation system. For registration the stepwise software instructions were followed. Briefly, the relevant vertebral level was defined by identifying three positions (right, left and center). These points identified the vertebra and its orientation. Subsequently, another 17 points were acquired on the same vertebra to map its surface. Registration was only performed on the vertebra where the dynamic reference frame was attached. The software then matched the acquired surface map to a 3D model created by segmenting the preoperative CT scan (performed automatically on the imported image set), to establish a coordinate system for navigation (Fig. 2). The registration accuracy was manually verified using the navigation pointer, and the registration was subsequently stored in the system.

### Automatic image registration using the Universal AIR device

**Fig. 3:**
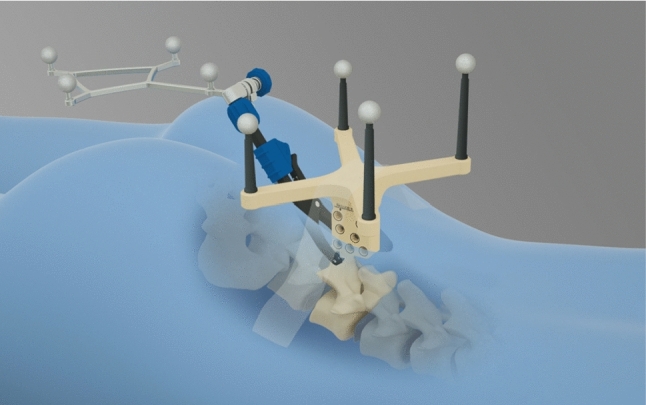
Schematic illustration of the clinical setup with the Universal AIR matrix for small incisions, the dynamic reference frame attached to the spinous process, both devices in proximity to the intended surgical level(s)

Universal AIR registration is a solution combining hardware and software designed for intraoperative 3D imaging-based registration in spinal and cranial surgery. By placing a registration matrix, containing radio-opaque and infrared markers, in the imaging field, the scanned volume is automatically registered for navigation, eliminating the need for additional imaging device integration (Fig. 3). This scanner-independent method provides immediate registration of intraoperatively acquired patient data from various CT, rotational angiography, and cone-beam CT systems. The registration matrix is positioned in the surgical field during the registration scan. The presence of the predefined matrix allows the image space to be registered to physical space for use by the navigation system; a linear transformation is calculated from the image coordinate system to the registration marker coordinate system and from the registration marker coordinate system to the dynamic reference frame coordinate system. Once the registration is completed, the registration matrix can be removed from the surgical field and patient tracking relies on the dynamic reference frame of the navigational system.

**Fig. 4:**
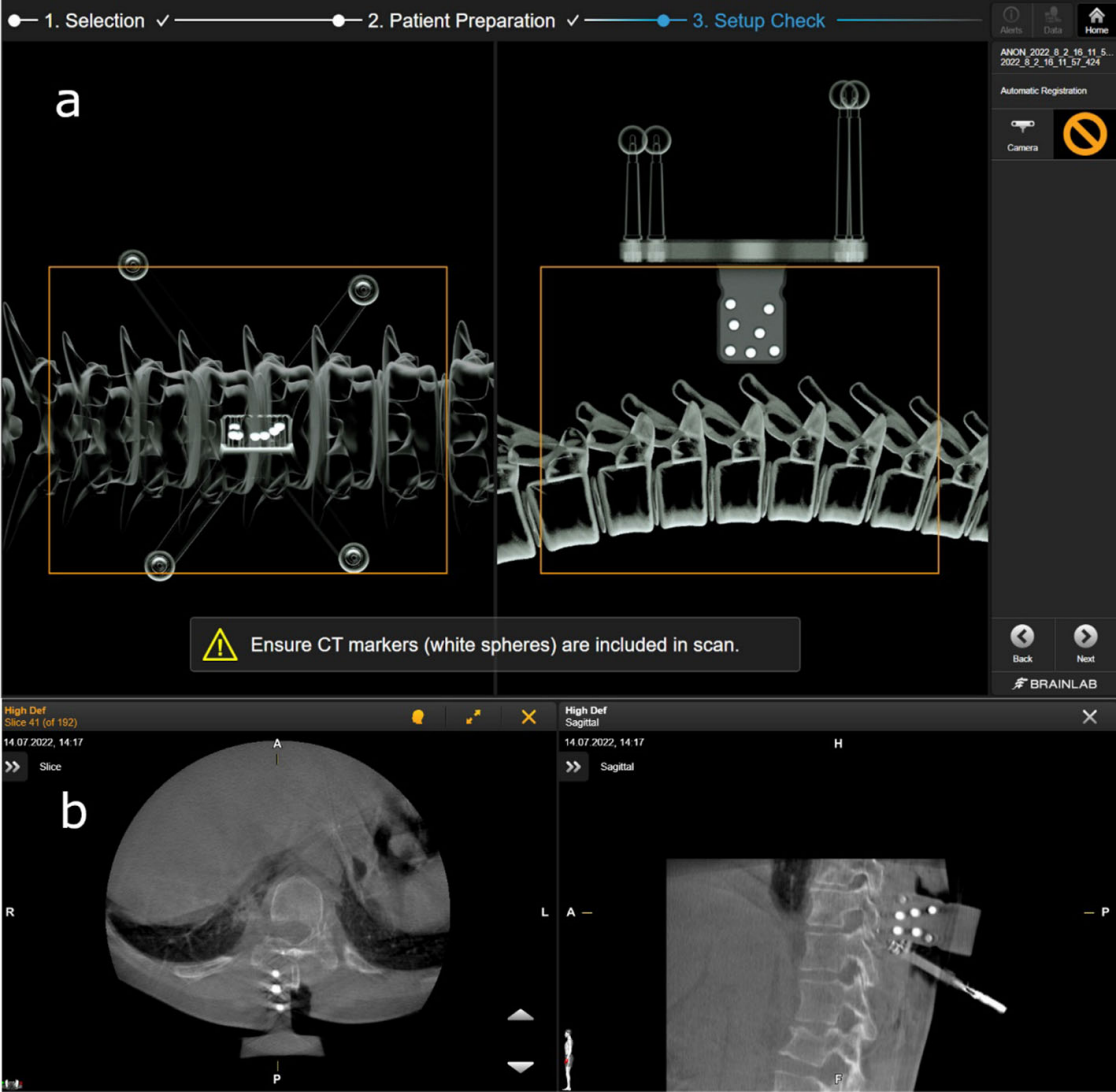
** a** Software representation of the universal AIR matrix with its combination of infrared and radio-opaque markers required for automatic registration. The box illustrates the scan volume. **b** CT images of the universal AIR matrix

Briefly, the Universal AIR matrix for small incisions was placed adjacent to the selected vertebra. Metallic retractors were removed to avoid image artifacts. Using the O-arm for fluoroscopy, all radiological markers of the registration matrix were made visible in both lateral and anteroposterior views. An intraoperative CBCT was then performed using the O-arm. The images were transferred to the navigation system. The registration accuracy was manually verified on anatomical landmarks as in the Surface Matching registration, and the plan was stored in the system (Fig. 4).

### Screw head acquisition

**Fig. 5:**
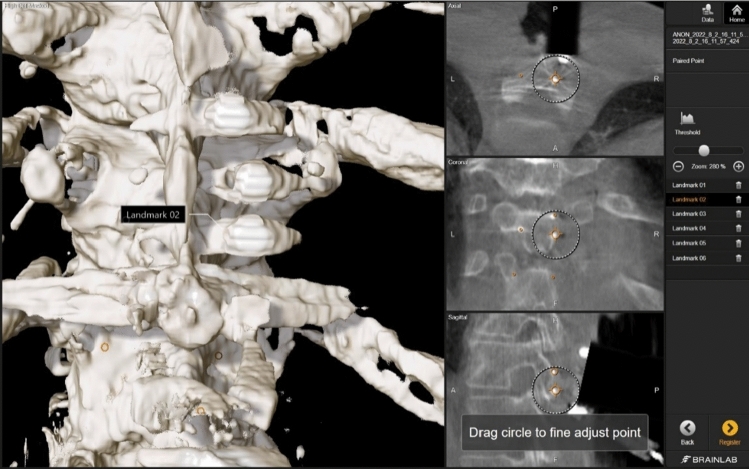
Definition and labeling of the “planned points” on the screw heads in the Software during the surgery

**Fig. 6:**
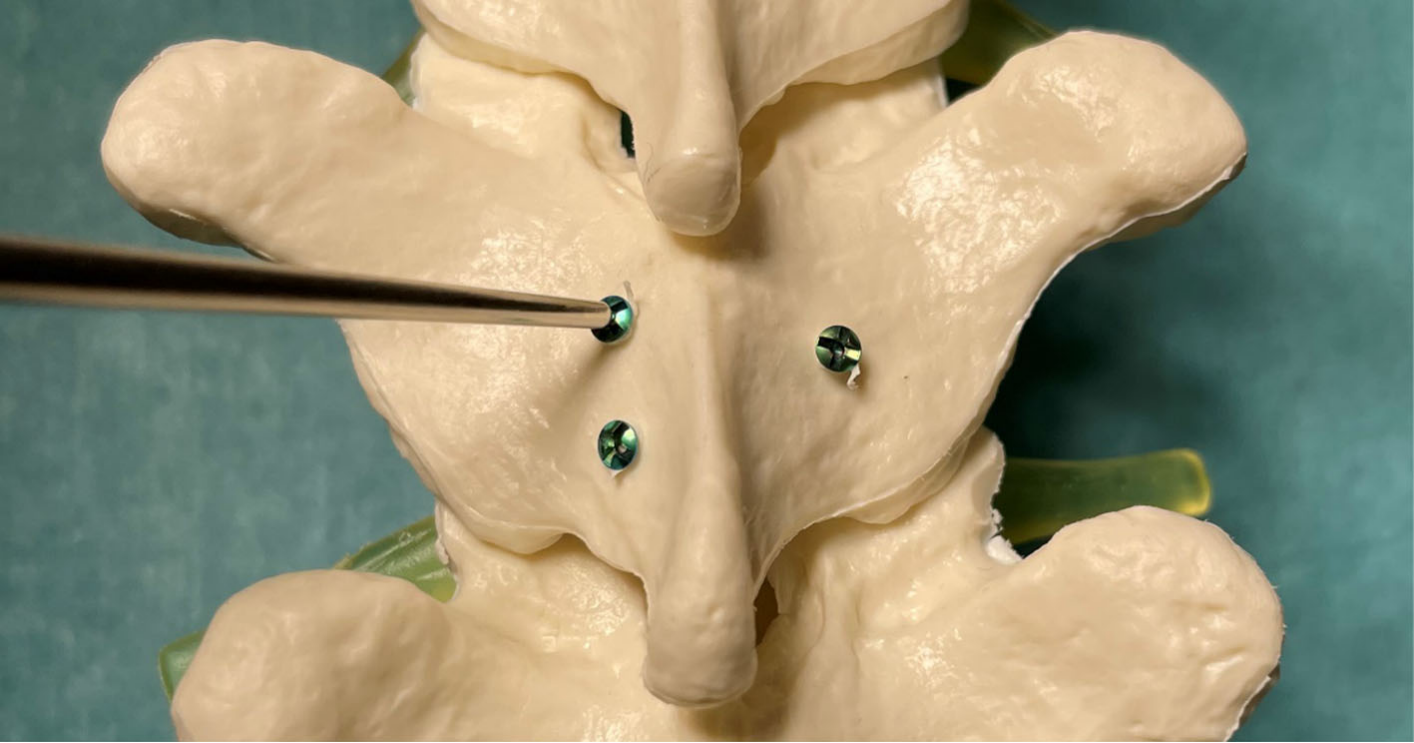
Illustration of how acquired points are obtained

Once the registration accuracy was accepted, the center positions of the three micro-screw heads were identified on the intraoperative CBCT scan, captured, and saved with a specified label in the software (planned screw head points) (Fig. 5). In addition, the centers of the three screw heads (where slits intersect to form a sort of crosshair) in physical space were captured with the navigation pointer and saved with a specified label in the software (acquired screw head points) (Fig. 6). The corresponding data, constituting a paired point matrix, were stored in the navigation system for later analysis and calculation of the accuracies (error)s of the two registration methods.

### Accuracy measurements

**Fig. 7:**
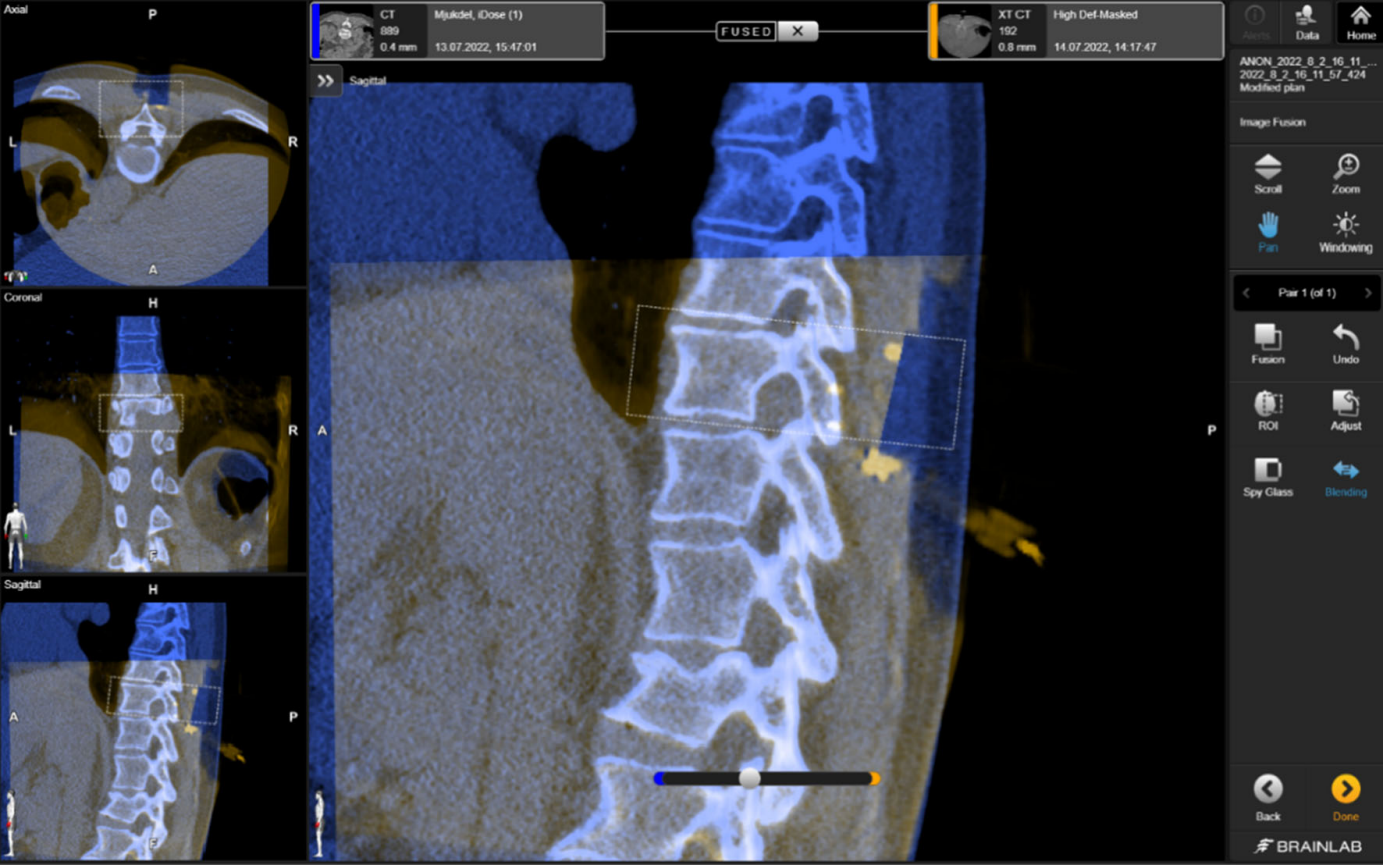
Image fusion of the vertebra of interest between the preoperative CT scan (blue) and the intraoperative CBCT scan (orange). Please note the visible bone screws on the lamina in the region of interest (orange)

**Fig. 8:**
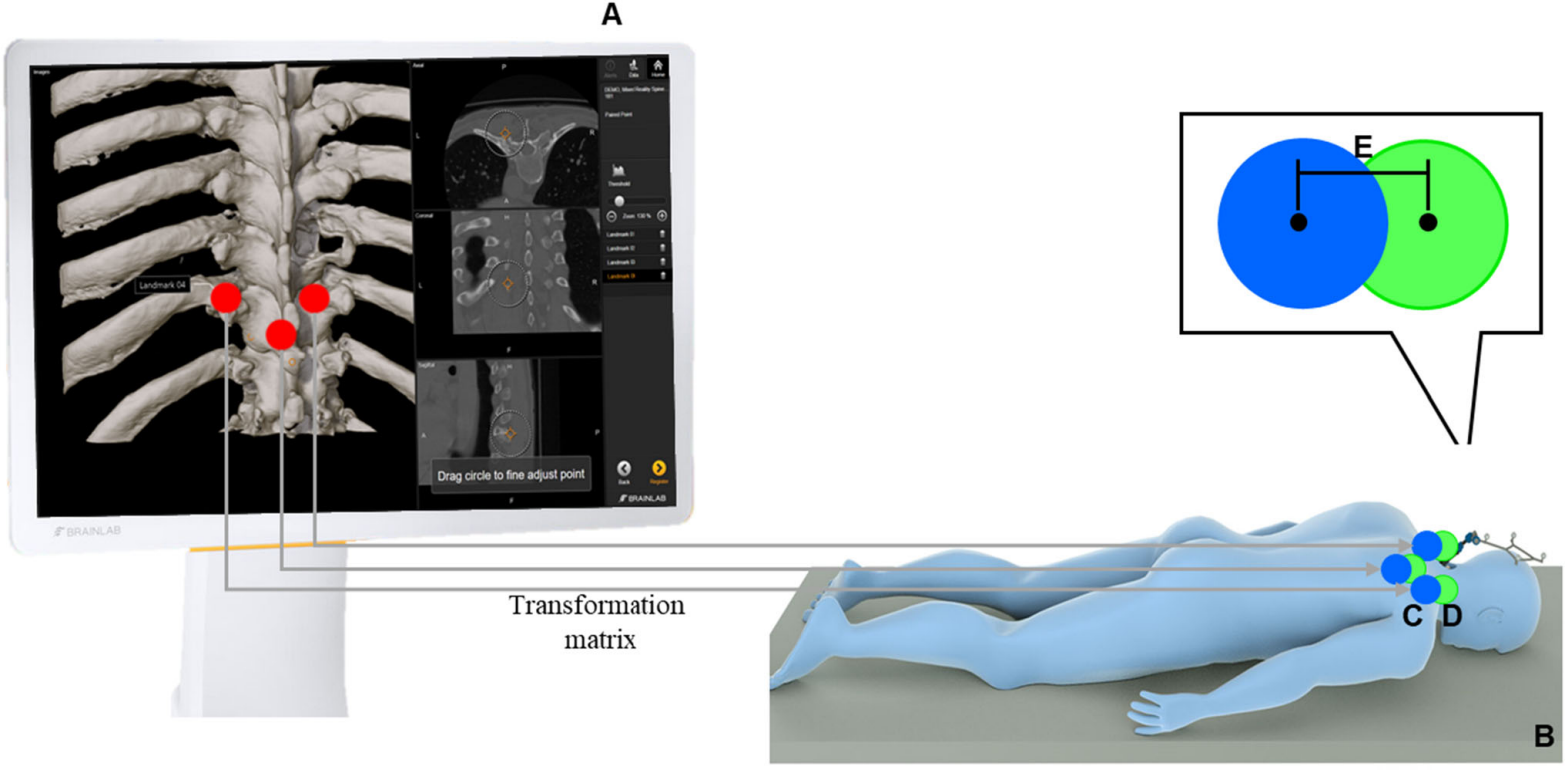
Error calculation method. After either Universal AIR or Surface Matching registration, the three implanted micro-screws were planned as points (in red) in the software (**A**). The coordinates of the planned screw points were then transformed from the intraoperative CBCT scan coordinate system to the patient reference coordinate system (**B**) to align the now transformed planned screw points (blue = transformed planned screw points) (**C**) with the acquired screw points on the actual patient (green = acquired screw points) (**D**). Finally, accuracy was assessed by calculating the discrepancy between the green (acquired) and blue (transformed) screw points (**E**) using the root mean square (RMS), as outlined in the materials and methods section

Registration accuracy was calculated by the transformation of points from one coordinate system to the other, to allow the subtraction of their coordinates in the same coordinate system. For the Universal AIR error calculation method, the planned screw points in the software were directly transformed from the intraoperative CBCT scan coordinate system to the patient reference coordinate system using the Universal AIR transformation matrix. For the Surface Matching method, the planned screw points were first transformed from the intraoperative CBCT scan coordinate system to the preoperative CT coordinate system using the fusion transformation matrix (Fig. 7); this was followed by the multiplication with the Surface Matching transformation matrix. This process aligned the now transformed planned screw points with the actual location of the screw points, which had been acquired on the patient using the pointer during the prior screw head acquisition step. Consequently, both sets of points were now within the patient reference coordinate system. The differences between the planned and the acquired screw point coordinates were considered the error. This transformation was executed for all points, and subsequently, the root mean square (RMS) was calculated to provide a comprehensive measure of the accuracy of the registration methods. An illustration depicting the transformation of points within the coordinate systems and the method of error calculation is shown in Fig. 8.

### Statistics

For the calculation of sample size, the values provided by Carl et al. [21] for automatic image registration with a mean value of 0.80 mm (± 0.28 mm SD) and by Zausinger et al. [22] with a mean value of 0.95 mm (± 0.3 mm) for Surface Matching registration have been used. Considering a significance level of *α* = 0.05 and a power of 0.9, a sample size of 42 patients for each registration method was required. The final sample size was calculated, with a 10% dropout rate, resulting in a total of 46 patients. However, during the data analysis phase, it was found that some inaccuracies and issues arose due to user errors. To account for these factors, a total of 50 datasets were collected. The normality of the data was assessed using the Shapiro–Wilk test which confirmed that the data followed a normal distribution (*p* > 0.05). A one-sided paired t test with a non-inferiority margin of 10% was performed to find out whether Universal AIR was non-inferior to Surface Matching registration. Furthermore, a post hoc paired-comparison t test was conducted to assess the difference between the measurement deviation of Universal AIR and Surface Matching registration methods. The threshold for statistical significance was established at 0.01.

## Results

### Patient inclusion and baseline characteristics

In total, 50 patients were eligible for inclusion. All patients had undergone a preoperative CT scan suitable for navigation. In three cases the intraoperative CBCT did not provide diagnostic quality, and these patients were excluded from further analysis. Among the remaining 47 patients, three patients with long constructs each contributed with two separate datasets from two different vertebral levels. Therefore, a total of 50 datasets were collected. Eight patients were excluded due to missing data, mismatches between the planned and acquired vertebral levels, inaccurate region match, or displacement of the dynamic reference frame. Thirty-nine patients with 42 complete and correct datasets were included in the final data analysis (Table 1).

**Table 1:** Patient characteristics

Number of patients included	39
Number of datasets	42
*Diagnoses*	
Cervical spinal stenosis	17 (40.5%)
Spinal schwannoma	10 (23.8%)
Spinal meningioma	5 (11.9%)
C1-C2 joint arthritis	3 (7.1%)
Revision surgeries	3 (7.1%)
Cervical fractures	2 (4.8%)
Other	2 (4.8%)
*Surgical procedure*	
Laminectomy/laminotomy	28 (66.7%)
Laminoplasty	10 (23.8%)
Posterior fixation	18 (42.9%)
Tumor excision	15 (35.7%)
Mean number of segments operated (SD)	3.0 (2.3)
*Spinal level*	
Cervical	20 (47.6%)
Cervicothoracic	13 (40.0%)
Thoracic	6 (14.3%)
Thoracolumbar	1 (2.4%)
Lumbar	2 (4.8%)

### Excluded patients

**Fig. 9:**
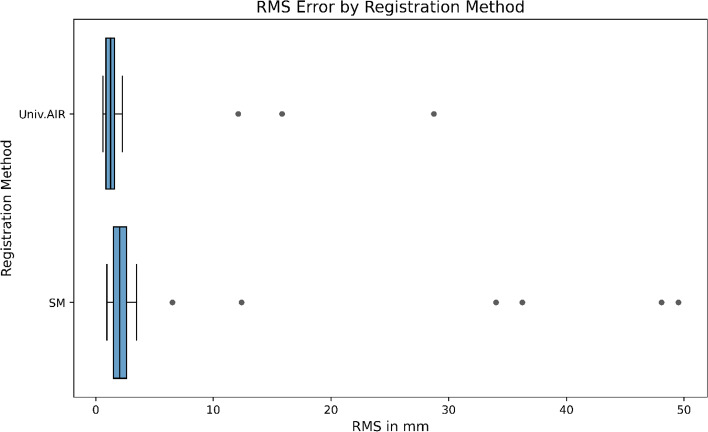
Accuracy comparison of registration methods: Box plot shows RMS in mm for 50 patients, with 8 outliers. SM method median score is 2 mm, with whiskers from 0.9 to 3.4 mm, but outliers reach up to 49.5 mm. Universal AIR method median score is 1 mm, with whiskers from 0.5 to 2.2 mm, with highest outlier at 28 mm

A total of 50 patients were included in the study. In three cases, the intraoperative scanner was not able to detect the Universal AIR matrix properly. This issue was solved for subsequent cases using the HD mode of the O-arm scanner to improve image quality. However, as there were no automatic image registration data available, these three patients were excluded from the study. Another eight patients were excluded from the final analysis due to user error or missing data. Their accuracy values can be seen as outliers in the box plot (Fig. 9, Supplementary Table 1).

### Technical accuracy

The Universal AIR registration reached a mean accuracy of 1.20 ± 0.42 mm and the Surface Matching registration reached a mean accuracy of 1.94 ± 0.64 mm. For Universal Air the maximum, minimum, and 99% confidence intervals were 2.23 mm, 0.60 mm, and 1.02 to 1.37. The corresponding data for Surface Matching registration were 3.45 mm, 0.92 mm, and 1.67 to 2.21. The difference in mean accuracy between Universal AIR and Surface Matching methods was calculated at − 0.74 mm (99% CI: − 1.03; -0 .46). The Universal AIR registration method was found to be non-inferior to the Surface Matching registration method (*p <* 0.0001). A post hoc analysis confirmed the greater accuracy of Universal AIR registration (*p <* 0.0001), as compared to the Surface Matching registration method.

**Fig. 10:**
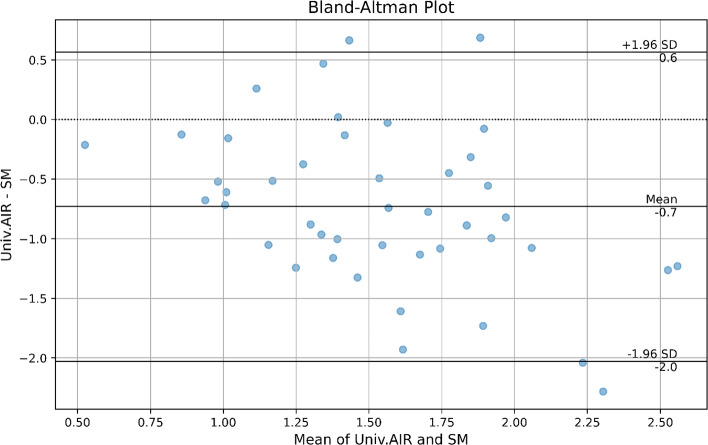
Bland–Altman plot demonstrating the agreement between Universal Air and SM Measurements. The mean difference is denoted by the central solid line, with the upper and lower solid lines representing the limits of agreement. The mean difference of -0.7 between the methods indicates that the error values of SM are greater than those of Universal Air

In assessing the level of agreement between Universal AIR and Surface Matching RMS values, a Bland–Altman plot was utilized (Fig. 10). The plot illustrates the differences between the RMS values obtained by the Universal AIR and Surface Matching methods against their means, highlighting the average bias and the limits of agreement. The Bland–Altman plot analysis indicates a mean difference (bias) of − 0.74, with the standard deviation of the differences being 0.68. The limits of agreement, which define the interval within which 95% of the differences between Universal Air and Surface Matching measurements lie, range from − 2.08 (lower limit) to 0.59 (upper limit). The absence of a proportional bias indicates that the methods agree equally through the range of measurements (i.e., the limits of agreement will not depend on the actual measurement).

## Discussion

The accuracy data of the present study demonstrate non-inferiority of automatic image registration using Universal AIR with intraoperative CBCT imaging as compared to the Surface Matching technique. In addition, a post hoc test revealed an improved registration accuracy using Universal AIR. In clinical terms, the accuracies of the methods, 1.20 mm and 1.94 mm, are both acceptable for spinal indications.

However, when using the Universal AIR automatic image registration, other benefits were noted. Significant outliers in accuracy values were noticed with the manual Surface Matching registration. The analysis of these cases revealed that user errors can occur leading to wrong level registration or incorrect point acquisition. This translates into failed or inaccurate registration. The exclusion of these cases from the analysis resulted in more consistent data, but still the distribution around the mean was narrower for the automatic registration.

Overall, we found that the use of Universal AIR together with the O-arm scanner was feasible. In some cases, insufficient image quality resulted in failed registration. However, setting the scanner mode to high definition (higher radiation dose with better image quality) allowed all registration scans to be successfully used for automatic image registration. The Universal Air matrix was easy to position in the surgical field and could rest suspended on the skin on either side of the incision. Three different sizes of the Universal AIR matrix are provided by the manufacturer, for different minimally invasive and open surgical scenarios. However, care must be taken that all the reflective spheres of Universal Air and the dynamic reference frame are visible to the IR camera of the navigation system during image acquisition. We experienced that the simultaneous detection of three different reference markers (the dynamic reference frame, the reflective spheres of the Universal Air and the radiopaque markers of the Universal Air) could be challenging, especially when the IR camera is looking “through” the O-arm gantry.

Using alternative methods for patient tracking could be a possible remedy for this difficulty. Most conventional navigation systems use a dynamic reference frame attached to the spinous process of a vertebra. The dynamic reference frame is normally shaped as a “star” with reflecting spherical markers that are recognized by an infrared camera. Although very effective for patient tracking and navigation, these reference frames are often bulky and occupy space in the vicinity of the surgical field, thereby interfering with the surgical procedure. They are also easily dislodged resulting in loss of navigational accuracy. To overcome these challenges, alternative tracking solutions have been introduced. Adhesive skin markers are used by the augmented reality surgical navigation system (ARSN) commercially known as ClarifEye (Philips, Best, The Netherlands), while Spine Mask (Stryker, Kalamazoo, MI, USA) utilizes LED lights in a frame attached to the patient’s back. Several augmented reality navigation devices use manual image superimposition [23], while sophisticated surface feature recognition methods have been proposed in experimental models [9, 24].

Integrated systems like the Loop X (Brainlab AG) paired with Brainlab navigation technology and the O-arm (Medtronic) paired with the StealthStation navigation technology utilize the infrared camera of the image-guided navigation system to detect reflective markers that are placed on the gantry of the scanner during the scan. The navigation software transforms the coordinate system of the 3D image to the coordinates of the patient and allows for immediate image registration and navigation without additional steps. A similar automatic registration method is used in ClarifEye, a hybrid OR-based navigation system for spine surgery where a robotic C-arm harbors the navigation cameras and registration occurs simultaneously during imaging [10].

Universal AIR instead provides a solution where non-integrated systems can be combined without losing automatic registration functionality. While Universal AIR offers the benefit of working with many intraoperative 3D imaging devices, it requires that the Universal AIR matrix and the region of interest (e.g., spine) are displayed in the same field of view (FoV) of the scanner. This can be challenging, particularly in obese patients, as the skin level with the attached matrix and the region of interest for surgery may be too distant from one another, making the use of the Universal AIR matrix registration problematic, particularly if a scanner has a limited FoV.

It has previously been shown that CBCT is as reliable as conventional CT for breach detection in spinal fixation surgeries [25]. Although the image quality provided by the O-arm (even when the HD mode is used) is inferior to conventional high-dose CT, the combination of Universal AIR automatic registration and O-arm scans provides excellent quality for intraoperative navigation and simultaneously making additional pose corrections superfluous. Preoperative CT scans are obtained with the patient in supine position and therefore require pose correction to accurately be used in navigation, with the patient in the prone position.

During the study, five cases of miss-matched vertebrae during Surface Matching occurred. This was particularly evident in thoracic cases where the vertebra of interest in the surgical field was incorrectly matched to an adjacent vertebrae on the preoperative CT scan. This wrong level matching and possible wrong level surgery is avoided when intraoperative imaging and automatic registration is used. In several cases where the fixation extended from C2-Th2, two separate CBCTs were obtained for instrumentation of upper cervical and upper thoracis levels. In contrast to navigation based on preoperative CT, by using automatic registration on intraoperative CBCT, accurate navigation was possible despite spine movements secondary to the instrumentation at one end of the surgical field.

### Strengths and limitations

This study was performed by surgeons with a long experience of different surgical navigation systems and patient registration methods. The results therefore reflect the clinical use of the technology in experienced hands but may not translate directly to inexperienced users or surgeons under training.

Navigation systems based on dynamic reference frames have been shown to lose accuracy with distance from the dynamic reference frame [10]. Since all measurements were performed on the same vertebra where the dynamic reference frame was attached, this phenomenon was not studied in this manuscript.

## Conclusions

Automatic image registration for spinal navigation using Universal AIR and intraoperative 3D imaging provided improved accuracy compared to Surface Matching registration. In addition, it minimizes user errors and offers a standardized workflow, making it a reliable registration method for navigated spinal procedures.

### Supplementary Information

Below is the link to the electronic supplementary material.

Supplementary file1 (DOCX 17 KB)

## Data Availability

Data are available from the corresponding author upon reasonable request.
