# Supplementary material for: Automatic image registration on intraoperative CBCT compared to Surface Matching registration on preoperative CT for spinal navigation: accuracy and workflow
Source: Int J Comput Assist Radiol Surg. 2024 Feb 20;19(4):665–75. doi: 10.1007/s11548-024-03076-4 (PMC10973038; doi:10.1007/s11548-024-03076-4)
Supplement: Supplementary file 1 — Supplementary file1 (DOCX 17 KB) [file 11548_2024_3076_MOESM1_ESM.docx]

Automatic image registration on intraoperative CBCT compared to surface matching registration on preoperative CT for spinal navigation: accuracy and workflow.

International Journal of Computer Assisted Radiology and Surgery

Henrik Frisk^1^, Gustav Burström^1^, Oscar Persson ^1^, Victor Gabriel El-Hajj^1^, Luisa Coronado^2^, Susanne Hager^2^, Erik Edström ^1,3^, Adrian Elmi-Terander^1,3,4^

^1^Department of Clinical Neuroscience, Karolinska Institutet, Stockholm, Sweden

^2^Clinical Affairs, Brainlab AG, Munich, Germany

^3^Capio Spine Center Stockholm, Löwenströmska Hospital, Upplands-Väsby, Sweden

^4^Department of Surgical Sciences, Uppsala University, Uppsala, Sweden

Correspondence:

Henrik Frisk, RN

Department of Clinical Neuroscience, Karolinska Institutet

171 77 Stockholm

henrik.frisk@ki.se

Supplementary Table 1 – Excluded cases and reason for exclusion

| Case # | Exclusion reason |
| --- | --- |
| 1 | Wrong SM points planned, resulting in the wrong level being matched by Surface Matching |
| 3 | Vertebra level mismatch: Surface Matching points were planned on L2 but acquired on L3 |
| 10 | Error in paired point matrix acquisition, which is the reference for the error calculation |
| 28 | Incorrect handling of the reference clamp:  - paired points were acquired on C2, while the patient reference array was placed on C1  - patient reference clamp was replaced after the Universal AIR scan and before the Surface Matching |
| 33 | Vertebra level mismatch:   - Surface Matching points were planned on L1 but acquired on L1: - Error in paired point matrix acquisition |
| 38 | Missing data |
| 44 | Vertebra level mismatch: patient reference array and fiducials for paired point were placed on L1, but Surface Matching was planned at T12 |
| 47 | Inaccurate region for Surface Matching by user accepted |
